# Supplementary material for: Production of a Novel Tetrahydroxynaphthalene (THN) Derivative from Nocardia sp. CS682 by Metabolic Engineering and Its Bioactivities
Source: Molecules. 2019 Jan 10;24(2):244. doi: 10.3390/molecules24020244 (PMC6358914; doi:10.3390/molecules24020244)
Supplement: Supplementary file 1 [file molecules-24-00244-s001.pdf]

Supplemental Materials for

Communication

Production of a Novel Tetrahydroxynaphthalene (THN) Derivative from *Nocardia* sp. CS682 by Metabolic Engineering and its Bioactivities

Ravindra Mishra [1,#], Dipesh Dhakal [1,#], Jang Mi Han [1], Haet Nim Lim [1], Hye Jin Jung [1,2], Tokutaro Yamaguchi [1,2] and Jae Kyung Sohng [1,2,*]

[1] Institute of Biomolecule Reconstruction (iBR), Department of Life Science and Biochemical Engineering, Sun Moon University, 70 Sun Moon-ro 221, Tangjeong-myeon, Asan-si, Chungnam 31460, Korea; ravindra.mishra44@gmail.com (R.M.); medipesh@gmail.com (D.D.); gkswkdal200@naver.com (J.M.H.); gotala9210@naver.com (H.N.L.); poka96@sunmoon.ac.kr (H.J.J.); yamaguchi@sunmoon.ac.kr (T.Y.)

[2] Department of BT-Convergent Pharmaceutical Engineering, Sun Moon University, 70 Sun Moon-ro 221, Tangjeong-myeon, Asan-si, Chungnam 31460, Korea.

[*] Correspondence: sohng@sunmoon.ac.kr; Tel.: 82-41-530-2246

# These authors contributed equally.

Figure Captions

Figure S1. Confirmation of deletion by PCR of (A) PCR probe for confirmation of deletion of PKS using probe 1 (~1.2kb) D1: *Nocardia* sp. CS682DR, W1: *Nocardia* sp. CS682 and probe 2 (~0.9kb) D2: *Nocardia* sp. CS682DR, W2: *Nocardia* sp. CS682, M: ladder; (B)PCR probe for amplification of marker gene *tsr*^{r} (1034 bp), M: DNA ladder, W: *Nocardia* sp. CS682, D: *Nocardia* sp. CS682DR (C) PCR probe for amplification of *apr*^{r} (777bp) V: pULVK2A used as positive control, M: DNA ladder, W: *Nocardia* sp. CS682, D: *Nocardia* sp. CS682DR

Figure S2. ^{1}H of NMR of compound IBR-3

Figure S3. ^{13}C NMR of compound IBR-3

Figure S4. COSY of compound IBR-3

Figure S5. ROESY of compound IBR-3

Figure S6. HSQC-DEPT of compound IBR-3

Figure S7. HMBC of compound IBR-3

Figure S8. HR-QTOF mass analysis of nargenicin A1

Figure S9. HR-QTOF mass analysis of compound IBR-1

Figure S10. HR-QTOF mass analysis of compound IBR-2

Figure S11. HR-QTOF mass analysis of compound IBR-3

Figure S12. HR-QTOF mass analysis of compound IBR-4

Figure S13. The anticancer activities of compound IBR-3 against various cancer cell lines

Figure S14. Melanin formation assay of compound IBR-3 using B16F10 melanoma cell

Table Legends

Table S1. List of strains and vectors used in the study

Table S2. Oligonucleotides used in the study

Fig. S1

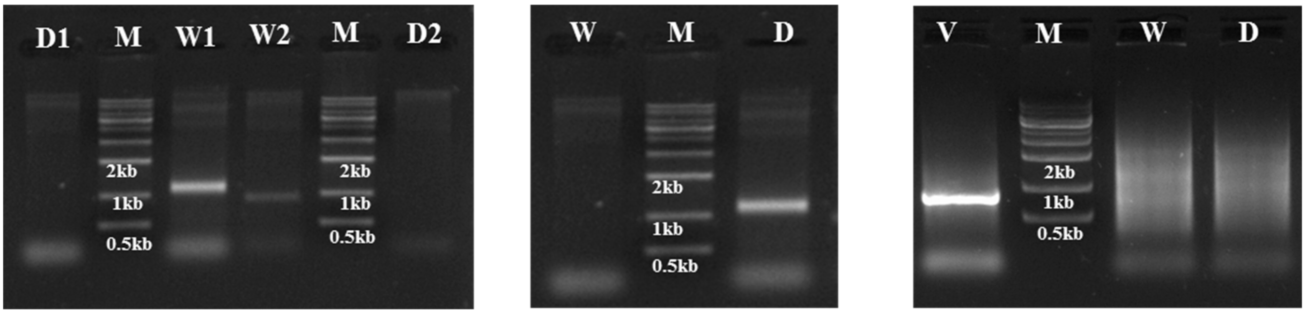
D1
M
W1
W2
M
D2
2kb
1kb
0.5kb
2kb
1kb
0.5kb
W
M
D
2kb
1kb
0.5kb
V
M
W
D
2kb
1kb
0.5kb

Fig. S2

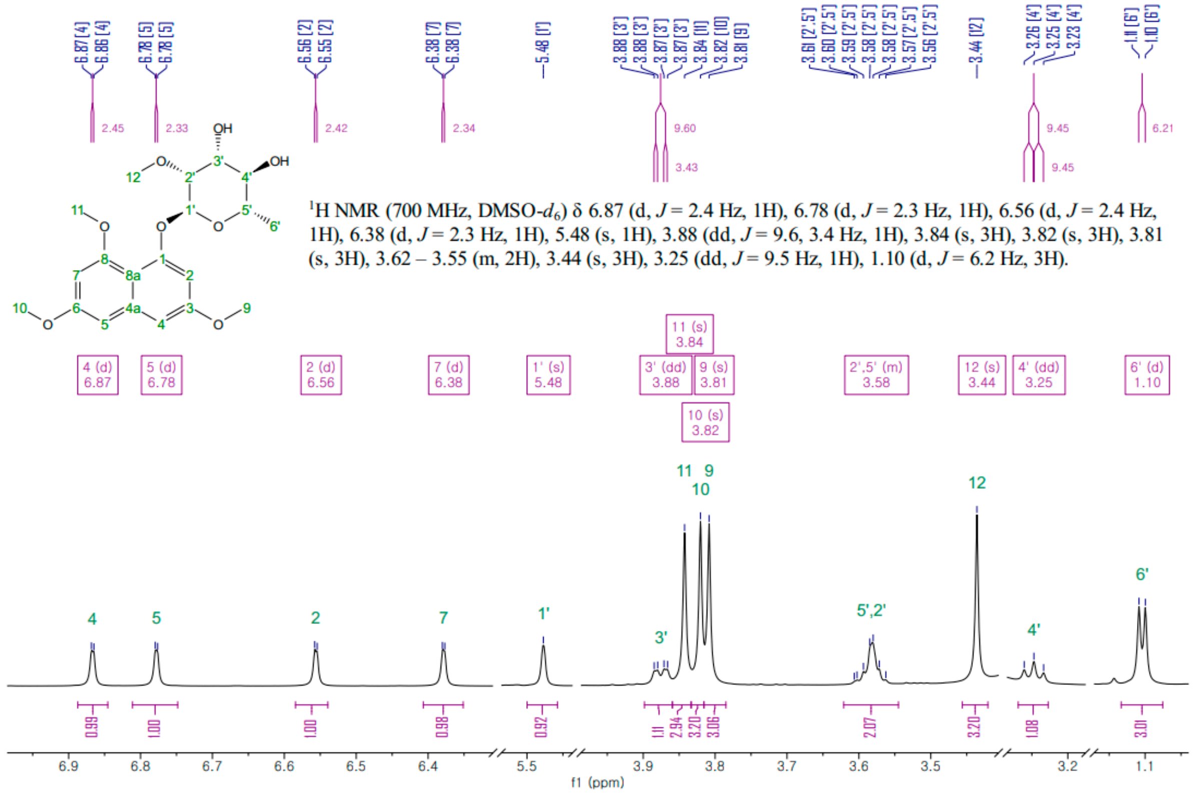
¹H NMR (700 MHz, DMSO-d_6) δ 6.87 (d, J = 2.4 Hz, 1H), 6.78 (d, J = 2.3 Hz, 1H), 6.56 (d, J = 2.4 Hz, 1H), 6.38 (d, J = 2.3 Hz, 1H), 5.48 (s, 1H), 3.88 (dd, J = 9.6, 3.4 Hz, 1H), 3.84 (s, 3H), 3.82 (s, 3H), 3.81 (s, 3H), 3.62 – 3.55 (m, 2H), 3.44 (s, 3H), 3.25 (dd, J = 9.5 Hz, 1H), 1.10 (d, J = 6.2 Hz, 3H).
f1 (ppm)

Fig. S3

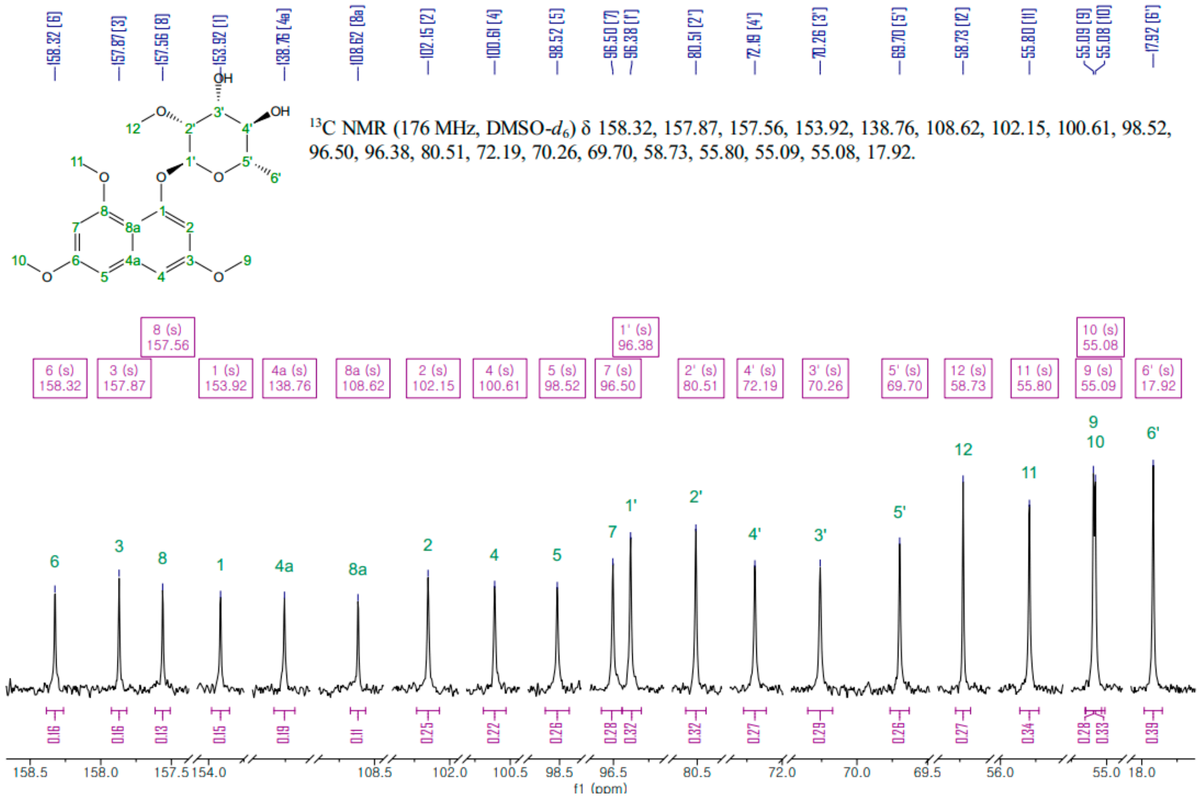

13C NMR (176 MHz, DMSO-d6) δ 158.32, 157.87, 157.56, 153.92, 138.76, 108.62, 102.15, 100.61, 98.52, 96.50, 96.38, 80.51, 72.19, 70.26, 69.70, 58.73, 55.80, 55.09, 55.08, 17.92.
f1 (ppm)

Fig. S4

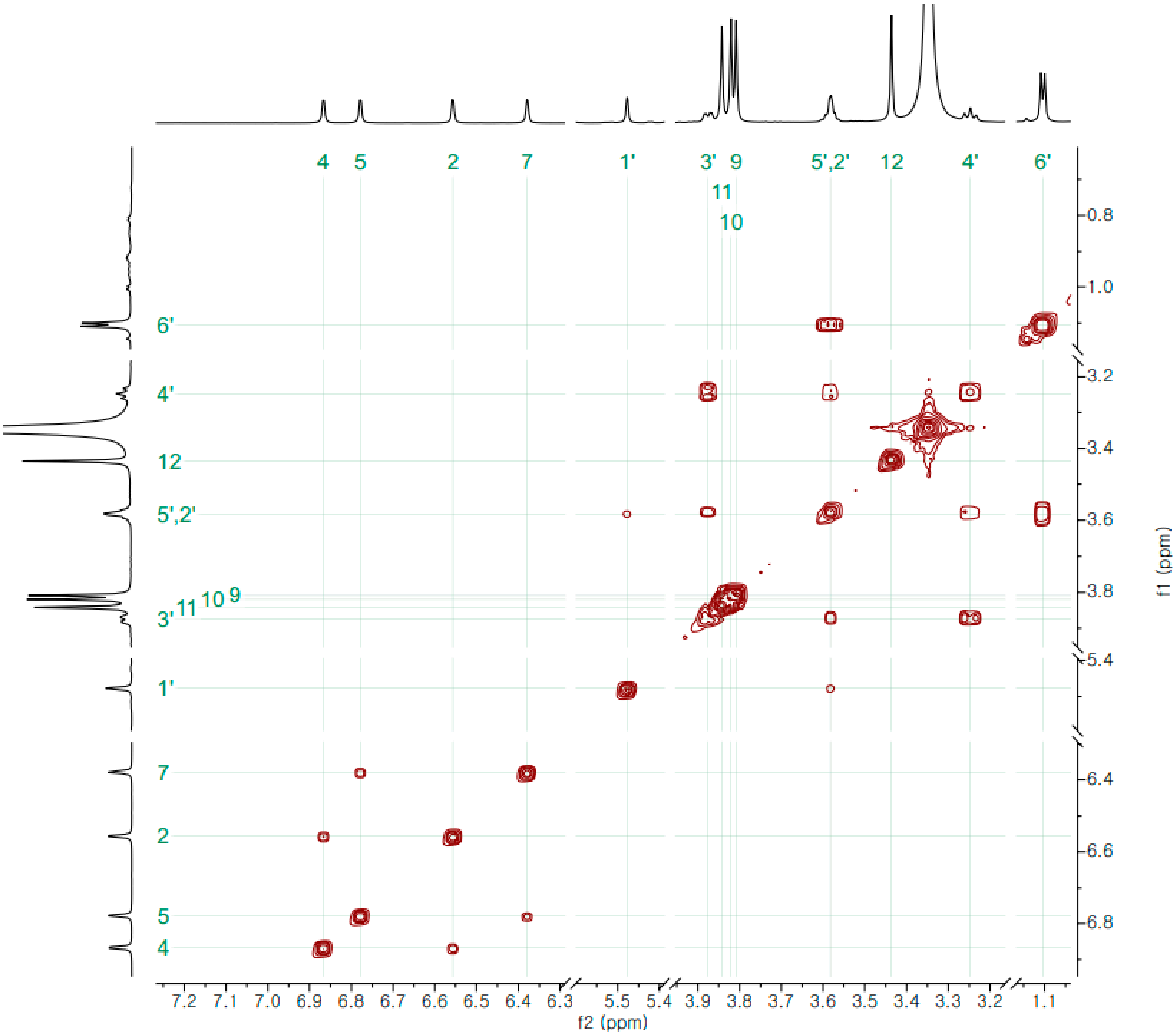

Fig. S5

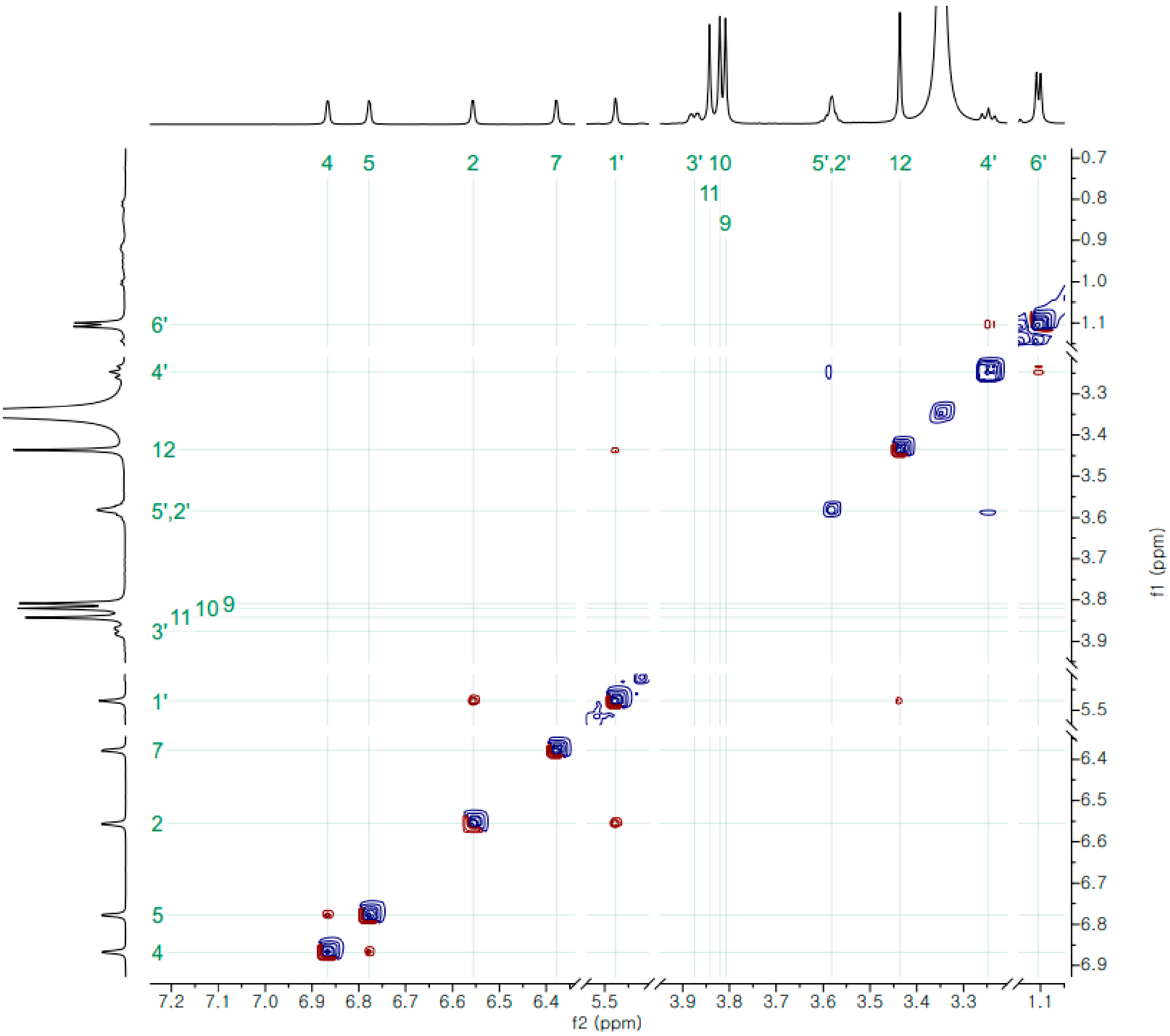

Fig. S6

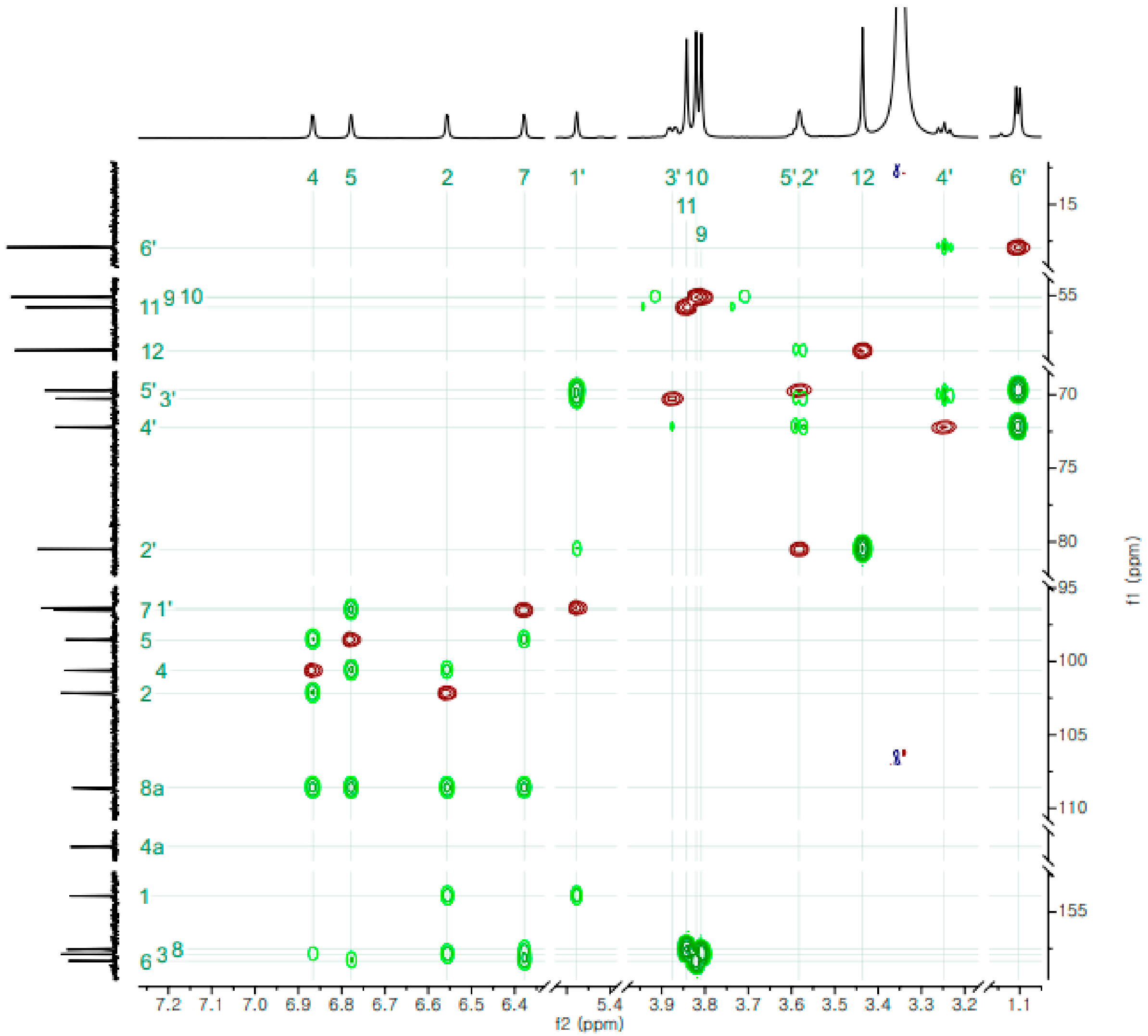

4
5
2
7
1'
3' 10
11
9
5',2'
12
4'
6'
6'
11 9 10
12
5' 3'
4'
2'
7 1'
5
4
2
8a
4a
1
6 3 8
15
55
70
75
80
95
100
105
110
155
f1 (ppm)
7.2
7.1
7.0
6.9
6.8
6.7
6.6
6.5
6.4
5.4
3.9
3.8
3.7
3.6
3.5
3.4
3.3
3.2
1.1
f2 (ppm)

Figure S7

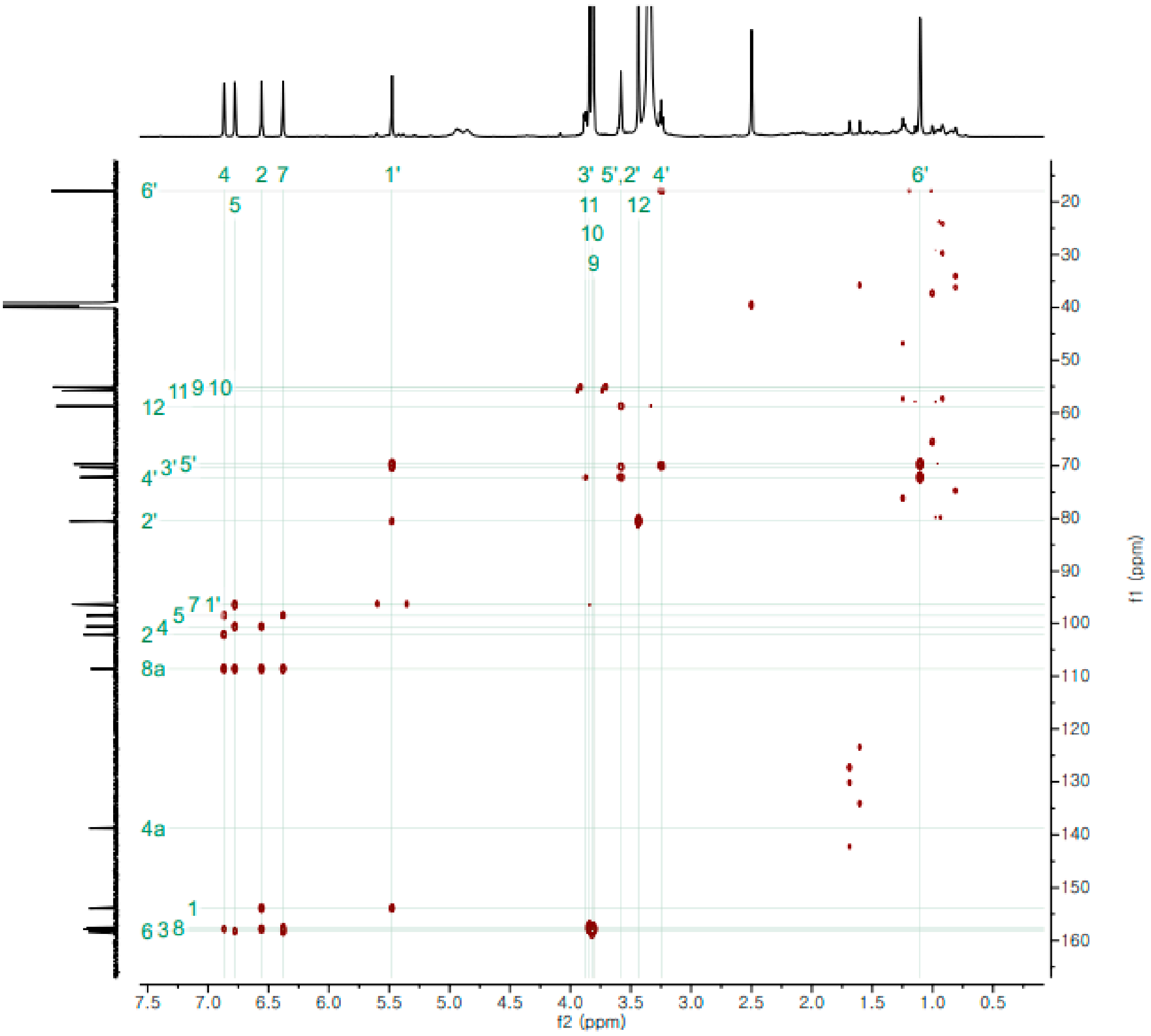
4
2
7
1'
3'
5'
2'
4'
6'
5
11
12
10
9
6'
12
11
9
10
4'
3'
5'
2'
5
7
1'
2
4
8a
4a
1
6
3
8
f2 (ppm)
f1 (ppm)

Fig. S8

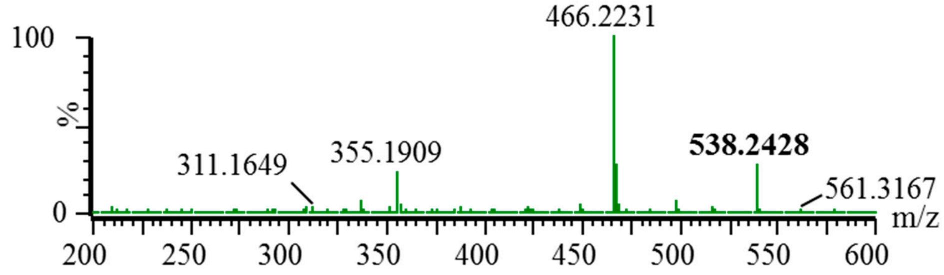
466.2231
100
%
0
311.1649
355.1909
538.2428
561.3167
m/z
200
250
300
350
400
450
500
550
600

Fig. S9

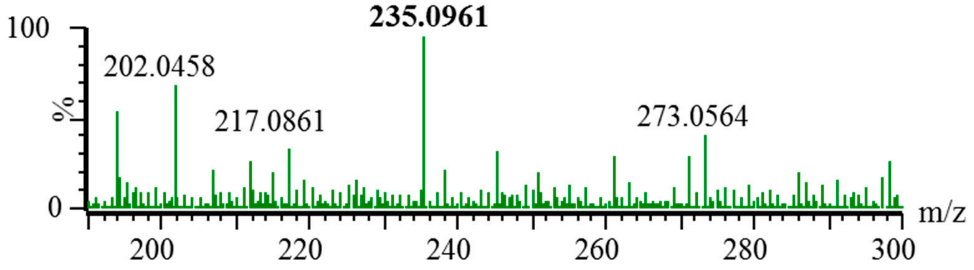
100
%
0
202.0458
235.0961
217.0861
273.0564
200
220
240
260
280
300
m/z

Fig. S10

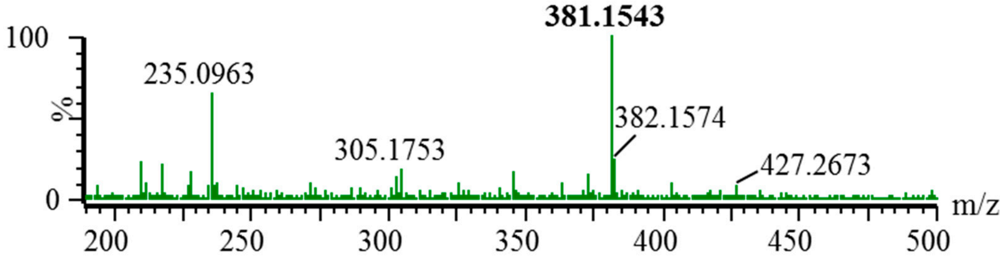
381.1543
100
235.0963
%
382.1574
305.1753
427.2673
0
m/z
200
250
300
350
400
450
500

Fig. S11

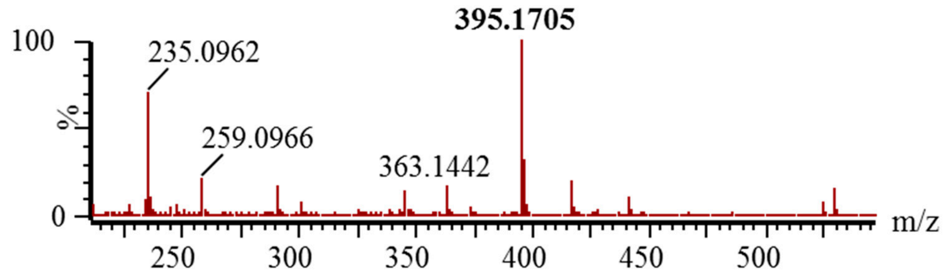
395.1705
100
235.0962
%
259.0966
363.1442
0
m/z
250
300
350
400
450
500

Fig. S12

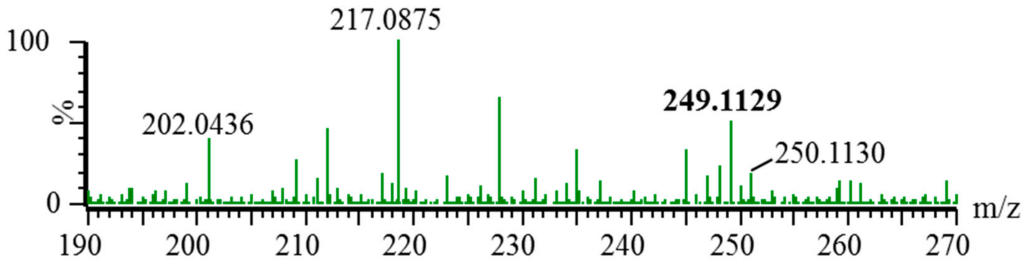

217.0875
100
202.0436
%
249.1129
250.1130
0
m/z
190
200
210
220
230
240
250
260
270

Figure S13.

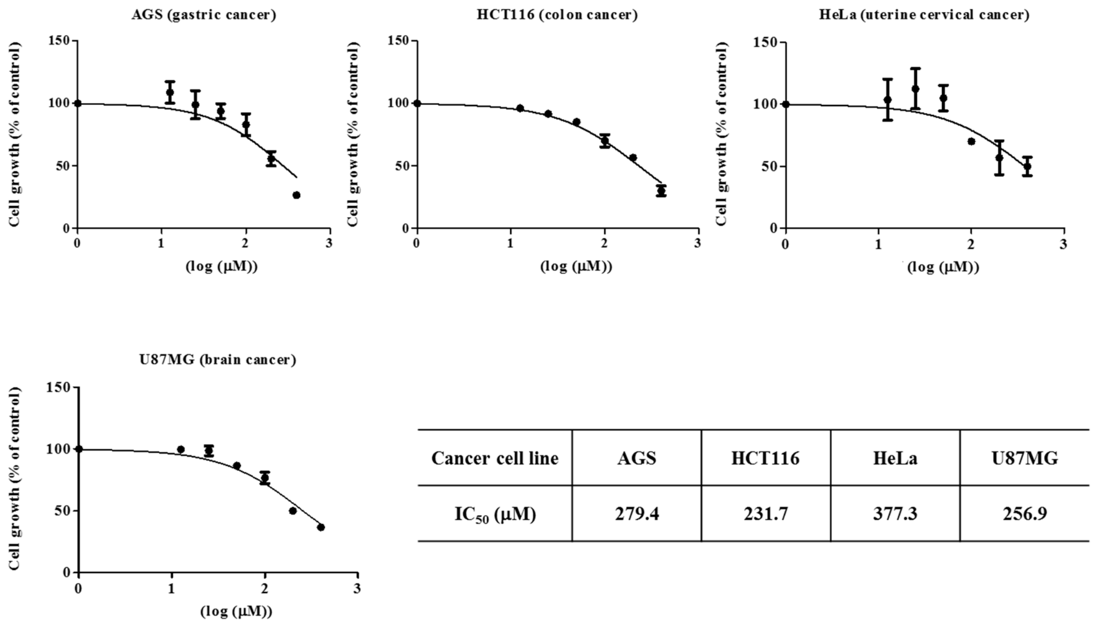

Cancer cell line	AGS	HCT116	HeLa	U87MG
IC_{50} (μM)	279.4	231.7	377.3	256.9

Figure S14.

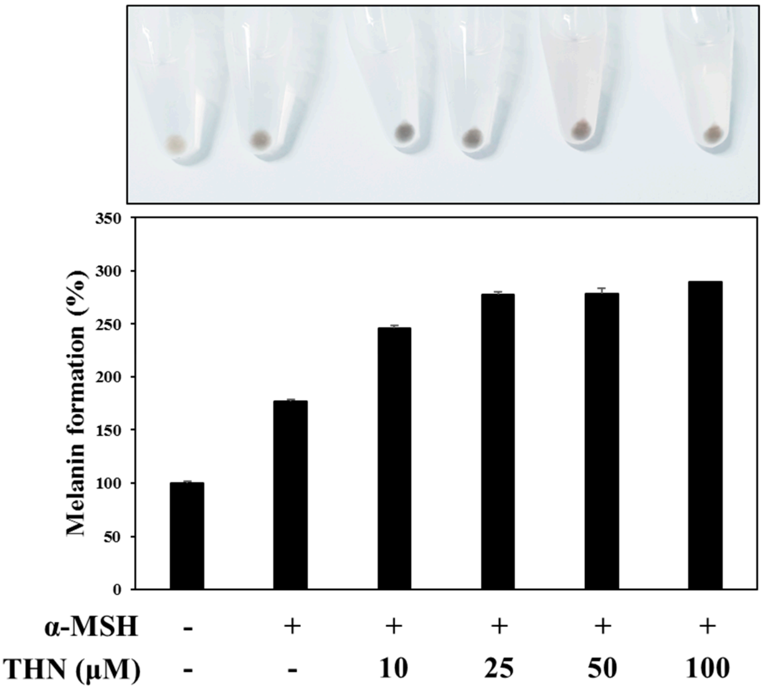
Melanin formation (%)
350
300
250
200
150
100
50
0
α-MSH
-
+
+
+
+
+
THN (μM)
-
-
10
25
50
100

Table S1. List of strains and vectors used in the study

Bacterial strains, plasmids and rDNAs	Relevant characteristics	Sources
**Bacterial strains**		
E. coli XL1Blue MRF	Δ*(mcrA)183* Δ(*mcrCB-hsdSMR-mrr)173 endA1 supE44 thi-1 recA1 gyrA96 relA1 lac*	Stratagene, USA
E. coli ET-12567	DNA demethylating strain (dam^- dcm^- hsdS cm^R)	John Innes Center, UK
Nocardia sp. CS682	Wild type producer of nargenicin A1	Sohng et al. 2008
Nocardia sp. CS682DR	Mutant strain with inactivation of nargenicin biosynthesis	This study
**Plasmids and rDNAs**		
pGEM®-T Easy	T_7 and SP6 promoters, *ColE1 ori, lacZ,* Amp^R	Promega, USA
pKC1139	Multi copy *E. coli-Streptomyces* shuttle vector	
pULVK2A	*Nocardia-E. coli* shuttle vector, neo^r, apr^r	Kelly and Townsend
pDN	pULVK2A containing deletion cassette for nargenicin A1 PKS region	This study.

Table 2. Oligonucleotides used in the study

Gene	Sequence (5′-3′)	Restriction site	Notes
delPKup-F	GGATTCGCCTCGCTGGACCGGGTC	*Bam*HI	For amplification of upstream region for deletion of PKS
delPKup-R	GGTACCGTCGGACGGTTCGTGCAA	*Kpn*I	
delPKdn-F	GGTACCGCTGGCCCGTGGACCACAGCG	*Kpn*I	For amplification of downstream region for deletion of PKS
delPKdnR	AAGCTTGTGCTGGTACTCCTGTGATCTCTCGGT	*Hind*III	
tsrF	GGTACCTGCAGTTAACTCTAGATTTAAATGTATGATCAAGGCGAA	*Kpn*I	For confirmation of double cross over mutant by PCR of thiostrepton resistance gene used as selection marker
tsrR	GGTACCTGCAGTTAACTCTAGATTTAAACAGAGGCGCTTATCGGT	*Kpn*I	
aprF	ATGCAATACGAATGGCGA		For confirmation of loss of vector in double cross over mutant by PCR of apramycin resistance gene
aprR	TCAGCCAATCGACTGGCG		
p1-F	CCCCGTACACCTTCACCGGGTCCC		For confirmation of deletion by PCR amplification of internal segment of PKS of nargenicin biosynthetic gene cluster
p1-R	GCCATGCCGACCCACTGCGAGCCCT		
p2-F	CGCCTCGCGTGCGTCGAGGACCGGA		
p2-R	GTCTTCTCACGGTCTCCTGAGCTGA		
orf1-F	CGAGCAAGCTATCCATCGTG		For qRT-PCR of *orf1*
orf1-R	ACAACGCCAGCATTATGACC		
orf2-F	GGTACGTTGCACCGTGATG		For qRT-PCR of *orf2*
orf2-R	CCTGCGAAATATTGCCTGCT		
thnM1-F	CGATGCTGGTGTTGACTGAG		For qRT-PCR of *thnM1*
thnM1-R	CGAATGGCCCAAATCGAACT		
thnT1-F	GCTCCTCGAAATGGACGATG		For qRT-PCR of *thnT1*
thnT1-R	CGCGTACAGCAGAATCTTGT		
thnG-F	CGGCTGGCCATGAGATATTG		For qRT-PCR of *thnG*
thnG-R	GACGATGCGAAAGCTAGTCC		
thnA-F	GAGTCAGGCTGGAACGAAA		For qRT-PCR of t*hnA*
thnA-R	ATCGTATGTGGGACTCGCTT		
thnM2-F	GTCAACACTGGTCTGACTGC		For qRT-PCR of *thnM2*
thnM2-R	TCGTGTCGATATCACCGGAT		
thnM3-F	CCAAATCGACACCGTAGCAG		For qRT-PCR of *thnM3*
thnM3-R	CTGGATCCCAGTTGTGGAGT		
thnT2-F	GTTTGTTGCTGACCGACCAT		For qRT-PCR of *thnT2*
thnT2-R	GGTGTTCGTATTGGGTCTGC		
orf3-F	TGATCGACAGAGCATTGGGT		For qRT-PCR of *orf3*
orf3-R	TGACCTCCGACGAAGTACAG		
16S-RT-F	AAAGAGCTTGTAGGCGGTCT		For qRT-PCR of *16S RNA*
16S-RT-R	TTCACCGCTACACCAGGAAT	-	
